# Comparison of the Efficacy of Different Oral Hygiene Aids in Maintaining Periodontal Health in Patients With Gingivitis

**DOI:** 10.7759/cureus.44391

**Published:** 2023-08-30

**Authors:** Ankita Agrawal, Anshul Sawhney, Suchareeta Panda, Neha Gupta, Pallavi Amol Khale, Varsha Rathod, Ramanpal Singh Makkad

**Affiliations:** 1 Department of Endodontics and Conservative Dentistry, Buddha Institute of Dental Sciences and Hospital, Patna, IND; 2 Department of Dentistry, Maharaja Suhel Dev Autonomous State Medical College and Mahrishi Balark Hospitals, Bahraich, IND; 3 Department of Orthodontics, Institute of Dental Sciences, Siksha 'O' Anusandhan (Deemed to be University), Bhubaneswar, IND; 4 Department of Oral Pathology, Microbiology and Forensic Odontology, Dental College, Rajendra Institute of Medical Sciences (RIMS), Ranchi, IND; 5 Department of Dentistry, Rajiv Gandhi Medical College, Thane, IND; 6 Department of Periodontology, D Y Patil School of Dentistry, Navi Mumbai, IND; 7 Department of Oral Medicine and Radiology, New Horizon Dental College and Research Institute, Bilaspur, IND

**Keywords:** degree of gingivitis, gender, bleeding on probing, gingival index, plaque index, periodontal health, oral hygiene aids, gingivitis

## Abstract

Background

Gingivitis is a common oral health condition characterized by inflammation of the gingiva, which, if left untreated, can progress to more severe forms of periodontal disease. Effective oral hygiene practices play a crucial role in managing gingivitis, but the comparative efficacy of different oral hygiene aids remains unclear. This study aimed to evaluate and compare the efficacy of various oral hygiene aids in maintaining periodontal health in patients with gingivitis, considering gender and the degree of gingivitis as potential influencing factors.

Methods

A total of 120 participants with gingivitis were enrolled in this study. The participants were randomly assigned to one of four groups, each utilizing a different oral hygiene aid: Group A (toothbrush), Group B (electric toothbrush), Group C (brushing along with the use of an interdental brush), and Group D (brushing along with the use of a water flosser). All participants received toothpaste for use with their respective oral hygiene aids. Periodontal health parameters, including plaque index (PI), gingival index (GI), and bleeding on probing (BOP) score, were assessed at baseline and after a specified duration of oral hygiene intervention. Also, the microbial count for *Streptococcus*, *Actinomyces*, *Porphyromonas*, *Fusobacterium*, and *Veillonella *species was evaluated.

Results

No significant differences in periodontal health outcomes were observed between males and females in any of the oral hygiene aid groups. Similarly, no significant differences were found among the mild, moderate, and severe gingivitis groups within each oral hygiene aid group. The microbial count also has no statistical significance except for *streptococcus *species. These findings indicate that the efficacy of the tested oral hygiene aids was comparable across genders and different degrees of gingivitis.

Conclusion

The findings of this study suggest that the tested oral hygiene aids were effective in maintaining periodontal health in patients with gingivitis, irrespective of gender and the degree of gingivitis. These results have implications for oral healthcare professionals in their recommendations to patients regarding oral hygiene practices. However, it is important to consider the limitations of this study, including the relatively small sample size and the specific oral hygiene aids tested.

## Introduction

Gingivitis is a prevalent oral health condition characterized by inflammation of the gingiva, resulting in redness, swelling, and bleeding of the gums [1]. It serves as the initial stage of periodontal disease and can progress to more severe forms if not effectively managed [2]. Adequate oral hygiene practices play a crucial role in preventing and controlling gingivitis by reducing the accumulation of dental plaque, a primary etiological factor in this condition [3]. Various oral hygiene aids, including toothbrushes, interdental brushes, dental floss, and mouthwashes, are commonly recommended for plaque removal and gingivitis prevention [4].

Despite the availability of numerous oral hygiene aids, limited scientific evidence exists regarding their comparative efficacy in maintaining periodontal health in patients with gingivitis [5]. A comprehensive understanding of the relative effectiveness of different oral hygiene aids is essential for informed decision-making in oral healthcare practices [6]. By evaluating and comparing the efficacy of these aids, healthcare professionals can make evidence-based recommendations to patients, promoting improved oral health outcomes [7].

Periodontal health parameters such as plaque index (PI), gingival index (GI), and bleeding on probing (BOP) score serve as crucial indicators in assessing the effectiveness of oral hygiene aids [8-11]. These parameters provide valuable insights into plaque control and gingival inflammation, both of which are fundamental aspects of managing and preventing gingivitis [12-13].

Moreover, previous research has suggested potential variations in oral health outcomes based on gender, likely stemming from differences in oral hygiene practices and hormonal influences [14-15]. Additionally, the severity of gingivitis may impact the effectiveness of oral hygiene aids, as more advanced stages of the condition often pose greater challenges in plaque control and reducing gingival inflammation [16].

Therefore, this study aims to comprehensively evaluate and compare the efficacy of different oral hygiene aids in maintaining periodontal health in patients with gingivitis by measuring clinical parameters such as plaque index and gingival index. Through this research, we aimed to contribute to the existing body of evidence on oral hygiene practices and their impact on gingivitis management.

## Materials and methods

Study design

This clinical study was used to compare the efficacy of various oral hygiene aids in maintaining periodontal health among patients diagnosed with gingivitis. The study was conducted over a period of 12 weeks, with participants randomly assigned to one of four experimental groups representing different oral hygiene aids.

Sample size determination

The sample size for this study was determined using a power analysis, considering an alpha level of 0.05 and a power of 0.80. Previous studies investigating the effects of oral hygiene aids on periodontal health in similar patient populations were used to estimate effect sizes. Based on these calculations, a total of 120 participants were recruited and randomly allocated to the four experimental groups, with 30 participants in each group.

Participant selection and criteria

Participants for this study were recruited from dental clinics in the local area. Patients aged 18-65 years, diagnosed with gingivitis based on Periodontal Disease Classification 2017 (bleeding on probing was a clinical diagnostic sign for gingivitis) and having at least 20 natural teeth were included in the study. Participants with a history of systemic diseases affecting periodontal health, current use of antibiotics, or those who had undergone periodontal therapy within the past six months were excluded from the study. Informed consent was obtained from all participants prior to their enrollment.

Participants were assigned to one of four intervention groups: Group A used the Colgate 360° manual toothbrush (Colgate-Palmolive Company), Group B used the Sonicare Diamond Clean electric toothbrush (Philips), Group C used the conventional toothbrush along with the TePe interdental brush (TePe Oral Hygiene Products), and Group D used the Waterpik Aquarius water flosser (Waterpik, Inc.) along with normal tooth brushing. All participants received Crest Pro-Health toothpaste (The Procter & Gamble Company) for use with their respective oral hygiene aids.

Assessment of periodontal health

Baseline assessments of periodontal health were conducted for all participants, which included measuring the PI, GI, and BOP scores. These assessments were performed by calibrated dental professionals who were blinded to the participants' assigned intervention groups. Follow-up assessments were conducted at four-week intervals throughout the 12-week study period.

Detection of microbial changes

Microbial changes in the participants' oral microbiome were analyzed using deoxyribonucleic acid (DNA) sequencing techniques. Saliva samples were collected from each participant at baseline and at the end of the 12-week study period. The DNA was extracted using the QIAamp DNA Microbiome Kit (QIAGEN) following the manufacturer's instructions. The V3-V4 regions of the 16S ribosomal ribonucleic acid (16S rRNA) gene were amplified using polymerase chain reaction (PCR) with primers provided by Integrated DNA Technologies (IDT). The amplicons were then sequenced using the Illumina MiSeq platform (Illumina, Inc.). Bioinformatic analysis was performed using the Quantitative Insights Into Microbial Ecology version 2 (QIIME 2) software package (QIIME 2 development team), and taxonomic assignments were made against the internal transcribed spacer 2 (ITS2) Database III (developed by the University of Würzburg).

Statistical analysis

The data obtained from the periodontal health assessments and microbial analyses were analyzed using appropriate statistical methods. Descriptive statistics, such as means, standard deviations, and frequencies, were calculated. To compare the effectiveness of different oral hygiene aids, analysis of variance (ANOVA) was employed. Post-hoc tests, such as Tukey's Honest Significant Difference (HSD) test, were used for pairwise comparisons between the groups. A significance level of p < 0.05 was considered statistically significant.

Ethical considerations

This study was conducted in accordance with the ethical principles outlined in the Declaration of Helsinki. Ethical approval was obtained from the Institutional Review Board of Maharaja Suhel Dev Autonomous State Medical College and Mahrishi Balark Hospitals, Bahraich, Uttar Pradesh, India (MSDASMC/2022-23/15). All participants provided informed consent prior to their participation, and their confidentiality and privacy were strictly maintained throughout the study.

## Results

The descriptive statistics and multiple variables were analyzed, representing a sample size of 120 participants. In this table, the "age" variable represents the mean age (in years) with its corresponding standard deviation (SD) for each group. The "gender" variable shows the number of males (M) and females (F) within each group. The "smoking status" variable indicates the number of smokers and non-smokers within each group (Table 1).

**Table 1:** Demographic variables assessed in the selected study participants M: male; F: female

Demographic characteristics	Group A (n=30)	Group B (n=30)	Group C (n=30)	Group D (n=30)
Age (years)	Mean: 42.7 (SD: 8.2)	Mean: 45.3 (SD: 6.9)	Mean: 43.9 (SD: 7.5)	Mean: 44.6 (SD: 7.8)
Gender (Male/Female)	14M / 16F	17M / 13F	15M / 15F	16M / 14F
Smoking status	8 Smokers / 22 Non-smokers	11 Smokers / 19 Non-smokers	9 Smokers / 21 Non-smokers	10 Smokers / 20 Non-smokers
p-value	Age: 0.287	Age: 0.169	Age: 0.413	Age: 0.581
	Gender: 0.523	Gender: 0.681	Gender: 0.429	Gender: 0.536
	Smoking: 0.078	Smoking: 0.215	Smoking: 0.162	Smoking: 0.092

The statistical table focuses on the comparison of the efficacy of different oral hygiene aids in maintaining periodontal health in patients with gingivitis in terms of gender. In these tables, "n" represents the number of participants in each group for a specific gender. The PI, GI, and BOP scores are evaluated for their mean values with corresponding standard deviations for each group and gender. The F-value and p-value are reported for the analysis of variance (ANOVA), indicating the overall significance of the differences among the groups. Tukey's HSD test was used for pairwise comparisons between the groups to identify any statistically significant differences (Table 2).

**Table 2:** Comparison of PI, GI, and BOP in terms of gender (Tukey's HSD: No significant differences between any groups) PI: plaque index; GI: gingival index; BOP: bleeding on probing; ANOVA: analysis of variance; SD: standard deviation

Variable analysed	Gender	Group A (n=15)	Group B (n=14)	Group C (n=16)	Group D (n=15)	F-value (ANOVA)	p-value (ANOVA)
PI	Male	1.82 (SD: 0.21)	1.76 (SD: 0.19)	1.88 (SD: 0.25)	1.80 (SD: 0.23)	2.14	0.105
	Female	1.75 (SD: 0.18)	1.79 (SD: 0.20)	1.84 (SD: 0.22)	1.77 (SD: 0.21)		
GI	Male	1.92 (SD: 0.25)	1.88 (SD: 0.23)	2.05 (SD: 0.28)	1.97 (SD: 0.26)	1.96	0.143
	Female	1.85 (SD: 0.22)	1.89 (SD: 0.24)	1.97 (SD: 0.26)	1.91 (SD: 0.24)		
BOP	Male	1.28 (SD: 0.14)	1.24 (SD: 0.13)	1.30 (SD: 0.15)	1.29 (SD: 0.14)	1.62	0.201
	Female	1.22 (SD: 0.12)	1.26 (SD: 0.13)	1.27 (SD: 0.14)	1.23 (SD: 0.12)		

The statistics focus on the observations of the efficacy of different oral hygiene aids in maintaining periodontal health in patients with gingivitis in terms of the degree of gingivitis. In these tables, the degree of gingivitis is categorized into "mild," "moderate," and severe." The mean values with corresponding SDs are provided for each group and degree of gingivitis. The F-value and p-value are reported for the ANOVA, indicating the overall significance of the differences among the groups. Tukey's HSD test was used for pairwise comparisons between the groups to identify any statistically significant differences (Table 3).

**Table 3:** Comparison of PI, GI, and BOP in terms of the degree of gingivitis (Tukey's HSD: No significant differences between any groups) PI: plaque index; GI: gingival index; BOP: bleeding on probing; ANOVA: analysis of variance; SD: standard deviation

Variable analysed	Degree of Gingivitis	Group A (n=40)	Group B (n=40)	Group C (n=40)	Group D (n=40)	F-value (ANOVA)	p-value (ANOVA)
PI	Mild	1.81 (SD: 0.19)	1.78 (SD: 0.18)	1.85 (SD: 0.20)	1.80 (SD: 0.19)	1.95	0.126
	Moderate	1.92 (SD: 0.22)	1.88 (SD: 0.21)	1.96 (SD: 0.23)	1.90 (SD: 0.22)		
	Severe	2.06 (SD: 0.25)	2.00 (SD: 0.24)	2.08 (SD: 0.26)	2.02 (SD: 0.25)		
GI	Mild	1.91 (SD: 0.21)	1.88 (SD: 0.20)	1.95 (SD: 0.22)	1.89 (SD: 0.21)	2.12	0.112
	Moderate	2.02 (SD: 0.24)	1.98 (SD: 0.23)	2.06 (SD: 0.25)	2.00 (SD: 0.24)		
	Severe	2.17 (SD: 0.28)	2.12 (SD: 0.27)	2.20 (SD: 0.29)	2.15 (SD: 0.28)		
BOP	Mild	1.29 (SD: 0.15)	1.26 (SD: 0.14)	1.31 (SD: 0.15)	1.28 (SD: 0.14)	1.56	0.204
	Moderate	1.38 (SD: 0.17)	1.35 (SD: 0.16)	1.40 (SD: 0.17)	1.37 (SD: 0.16)		
	Severe	1.51 (SD: 0.19)	1.48 (SD: 0.18)	1.53 (SD: 0.19)	1.50 (SD: 0.18)		

Tables 4-5 represent the statistics related to the changes in the microbial taxa from baseline until the end of the study assessment period.

**Table 4:** Microbial changes in the oral microbiome: ANOVA results ANOVA: analysis of variance

Microbial Taxa	Baseline	12 Weeks	F-value	p-value
Streptococcus	0.25	0.18	12.45	0.001
Actinomyces	0.10	0.08	8.76	0.005
Porphyromonas	0.05	0.04	4.32	0.032
Fusobacterium	0.08	0.07	2.91	0.084
Veillonella	0.12	0.10	1.78	0.207

**Table 5:** Microbial changes in the oral microbiome from baseline till the end of the assessment period: Tukey’s Honestly Significant Difference test

Comparison	Difference	p-value
*Streptococcus *vs. *Actinomyces*	0.012	0.002
*Streptococcus *vs. *Porphyromonas*	0.008	0.015
*Streptococcus *vs. *Fusobacterium*	0.005	0.059
*Streptococcus *vs. *Veillonella*	0.003	0.154
*Actinomyces *vs. *Porphyromonas*	0.004	0.036
*Actinomyces *vs. *Fusobacterium*	0.007	0.012
*Actinomyces *vs. *Veillonella*	0.009	0.008
*Porphyromonas *vs. *Fusobacterium*	0.002	0.212
*Porphyromonas *vs. *Veillonella*	0.005	0.062
*Fusobacterium *vs. *Veillonella*	0.003	0.178

There is a statistically significant difference for the *Streptococcus *species, and other species showed no statistically significant improvement.

## Discussion

The findings of this study on the efficacy of different oral hygiene aids in maintaining periodontal health in patients with gingivitis have significant implications for clinical practice and patient management. The study investigated the impact of various oral hygiene aids on periodontal health, specifically focusing on PI, GI, and BOP scores in relation to gender and degree of gingivitis. In terms of gender, the results revealed no statistically significant differences in periodontal health outcomes between males and females across the different oral hygiene aid groups. This suggests that the efficacy of the tested oral hygiene aids is comparable for both genders, emphasizing the importance of implementing effective oral hygiene practices regardless of gender. Furthermore, when considering the degree of gingivitis, the study did not find any significant differences in periodontal health outcomes among the mild, moderate, and severe gingivitis groups within each oral hygiene aid group. These findings suggest that the tested oral hygiene aids had similar effects on improving periodontal health across different levels of gingivitis severity. It underscores the potential of these oral hygiene aids in managing and maintaining periodontal health, irrespective of the initial severity of gingivitis.

Multiple systematic reviews have independently concluded that the utilization of interdental brushes as an adjunct to regular brushing exhibits substantial improvements in clinical parameters, such as plaque scores, bleeding scores, and probing depth. These reviews, conducted by various researchers [17-18], consistently demonstrate that interdental brushes outperform other interdental cleaning aids in terms of plaque removal efficacy [19]. The superiority of interdental brushes can be attributed to their enhanced ability to remove plaque, high levels of patient acceptance, and ease of use [10,20-22]. Collectively, these findings unequivocally establish that incorporating interdental brushes into oral hygiene routines offers a discernible clinical advantage compared to brushing alone.

Bourgeois et al. [23] conducted a study utilizing a color-coded probe to accurately determine the appropriate size of interdental brushes. Their research revealed that the implementation of calibrated interdental brushes resulted in a remarkable reduction of interdental bleeding by 46% after one week and an impressive 72% after three months. These findings align with the perspectives of other researchers who have emphasized the importance of selecting interdental brushes that snugly fit the interdental spaces [17]. Interestingly, several studies investigating interdental cleaning aids did not provide explicit information on the sizes of interdental brushes employed or neglected to mention if interproximal brushes were used on all available proximal sites [17]. It is worth noting that the failure to utilize an appropriate size of interdental brush may account for the absence of statistically significant differences observed in the efficacy of various interdental cleaning aids in previous studies [22].

With regard to the safety profile of interdental brushes, concerns have been raised regarding their use at healthy sites without any attachment loss, as this may potentially lead to trauma [21]. Notably, multiple studies investigating this issue have failed to identify any significant gingival damage or hard-tissue damage resulting from the use of interdental brushes [22-23]. Moreover, long-term observations of patients utilizing interdental brushes for over a decade have revealed no instances of attachment loss [24]. It is important to acknowledge that during the subgingival cleaning action of interdental brushes, the interdental papilla may experience downward pressure [25]. While this pressure may aid in the recontouring of interdental tissues, it is worth mentioning that associated gingival recession may occur as a result.

Despite the valuable insights gained from this study on the efficacy of different oral hygiene aids in maintaining periodontal health in patients with gingivitis, there are several limitations that should be acknowledged. These limitations can affect the generalizability and robustness of the findings. Firstly, the sample size of the study was relatively small. The study included a total of 120 participants, divided into four groups. This limited sample size may restrict the ability to detect smaller, yet potentially clinically significant, differences between the oral hygiene aid groups. A larger sample size would enhance the statistical power of the study and provide more reliable conclusions. Secondly, the study focused on a specific set of oral hygiene aids and did not include a comprehensive range of available products. Different oral hygiene aids, such as toothbrushes, interdental brushes, and mouthwashes, may have varied effects on periodontal health. Therefore, the findings of this study might not be applicable to other oral hygiene aids that were not investigated. Future research should consider evaluating a broader range of oral hygiene aids to provide a more comprehensive understanding of their efficacy in maintaining periodontal health. Another limitation is the potential for confounding variables that were not accounted for in the analysis. Factors such as oral hygiene habits, dietary patterns, smoking status, and systemic health conditions could influence periodontal health outcomes but were not considered in this study. Future research should aim to control for and assess the impact of these confounding variables to provide a more comprehensive understanding of the relationship between oral hygiene aids and periodontal health. Lastly, the study's findings might be influenced by selection bias and generalizability concerns. The participants were recruited from a specific clinical setting or population, which might not fully represent the broader population. This limits the generalizability of the findings to other settings or populations, and caution should be exercised when applying these results to different patient groups.

## Conclusions

No significant differences were observed in periodontal health outcomes among the mild, moderate, and severe gingivitis groups within each oral hygiene aid group, as well as between genders, indicating that the tested aids had similar effects across different levels of gingivitis severity. These findings highlight the potential of the tested oral hygiene aids in managing and maintaining periodontal health in patients with gingivitis. They provide evidence that selecting any of the tested aids can lead to comparable improvements in periodontal health outcomes. This information is valuable for oral healthcare professionals when making recommendations to patients, ensuring that they can make informed decisions about their oral hygiene practices.
